# A Qualitative Study of Barriers to Personal Protective Equipment Use among Laundry Workers in Government Hospitals, Hawassa, Ethiopia

**DOI:** 10.1155/2020/5146786

**Published:** 2020-09-22

**Authors:** Aiggan Tamene, Abel Afework, Lewam Mebratu

**Affiliations:** ^1^Department of Public Health, College of Medicine and Health Sciences, Wachemo University, Hosaena, Ethiopia; ^2^Dilla University Referral Hospital, Dilla University, Dila, Ethiopia; ^3^Hawassa University Comprehensive Specialized Hospital, Hawassa University, Awasa, Ethiopia

## Abstract

**Background:**

The need to reduce the transmission of infectious diseases makes the use of personal protective equipment and safety medical devices compulsory among hospital laundry staff. The practice, however, remains to be low among hospital laundry staff members. Globally, not many studies seem to have been carried out to sufficiently tell us about the barriers to personal protective equipment use among hospital laundry workers. Related studies in Ethiopia are even fewer. This study assessed the barriers to personal protective equipment use among laundry staff of government hospitals in Hawassa City, Southern Ethiopia, 2019.

**Methods:**

Two qualitative data-gathering methods—focus group discussions and key informant interviews—were used to collect data for this study. Eight focus group discussions were conducted with hospital laundry workers. Similarly, six key informant interviews were held with Infection Prevention and Patient Safety Officers. Thematic analysis was performed using Open Code 4.02.

**Result:**

Organizational- and individual-level barriers such as unavailability of essential personal protective equipment, a disharmonious work environment, low perception of susceptibility, and belief about personal protective equipment interference with work performance were identified as the major barriers to personal protective equipment use in the present study.

**Conclusion:**

Organizational- and individual-level barriers have been identified as causes for the low level of personal protective equipment use among hospital laundry workers. Therefore, improving institutional supplies in quantity and quality may have a positive implication for the improvement of infection prevention practices in the study area. Also, designing sustainable strategies and raising laundry workers' awareness of a safe work environment may lead to the improvement of infection prevention practices.

## 1. Introduction

Personal protective equipment (PPE) is a device designed to create physical barriers between the worker and workplace hazards to protect the worker against work-related injuries and illnesses [1]. In the health care setting, personal protective equipment is mainly used to protect the health care personnel from the exposure of pathogens especially Health Care Acquired Infections (HAIs) [2]. The use of PPE is essential in Infection Control (IC). It protects laundry staff from acquiring dangerous infections and diseases of epidemic proportions. However, compliance with universal precautions among workers is poor even in the face of high-risk clinical situations [1].

Hospital laundries are in charge of distributing disinfected linen throughout several units of a hospital. This is a very crucial activity in a health care institution, as either lack of or delayed distribution of hospital linens affects the activities of the hospital and influences the quality of health care [3]. Operating rooms, in-patient units, intensive care units (ICUs), out-patient wards, etc. strongly depend on the proper performance of the laundry service. This means that a problem in the distribution of hospital linens may lead to more serious problems in patients' care and even in scheduled activities such as surgeries and hospital stays [4]. Hospital laundries are, therefore, very important in preventing hospital-acquired infections.

Despite the importance of this service, there is little concern for the workers' safety and health in many hospitals [5]. Hospital laundry workers have strenuous schedules and are exposed to different occupational and environmental hazards. These workers are exposed to physical and chemical hazards such as heat, humidity, vibration, dust, smoke, gas, and noise. They also risk punctures and lacerations caused by needles and sharp objects. Infections caused by microorganisms are yet other dangers facing hospital laundry workers [5]. The psychological toll from the productivity demands in their work setups should also not go unnoticed [6].

A large number of factors have been identified as barriers to PPE use in prior studies in this area. Equipment unavailability, lack of safety training, workers' perceived lack of time to wear PPE, and pressure from colleagues have been reported to be significant barriers to PPE use among hospital laundry staff. Similarly, fear of compromising productivity was also reported as an important PPE use hindering factor among hospital laundry workers [5, 7, 8].

Ethiopia envisions reaching universal health coverage by 2035. Evidence of realizing this can be found in the efforts currently being made to raise the number of hospitals in the country to 800 [9]. At present, there are 229 hospitals in the country. However, despite the presence of such a fairly large number of hospitals, there is limited information on barriers to PPE use among hospital laundry workers in the local context. This study was, therefore, designed to explore barriers to hospital laundry workers' use of PPE in Hawassa City, Ethiopia.

## 2. Methods and Materials

The study was conducted in two government hospitals in Hawassa City from March 25 to April 22, 2019. The two government hospitals were purposively selected for this study because of their large patient flow and the high productivity demand prevalent in their laundry departments.

The study team consisted of three main researchers, four health and safety professional data collectors, and one experienced supervisor. A day-long intensive training was given to the supervisor and the four data collectors. During the training, lessons on data-gathering tools in the study and how the study participants could best be approached were discussed in detail.

The study had two groups of participants. One group consisted of workers from the laundry departments, while participants in the other group were Infection Prevention and Patient Safety Officers within the hospitals. Infection Prevention and Patient Safety Officers were environmental and occupational health professionals who were in charge of dispensing personal protective equipment to hospital staff. They were also responsible for supervising the work of laundry and cleaning departments. To select the study participants, an on-site census was conducted in both hospitals. During the on-site census, a total of 80 workers were recognized as operating in the laundry departments. The Infection Prevention and Patient Safety Offices had nine staff members.

The design of this study is qualitative. The data-gathering instruments used in this study were semistructured key informant interviews (KII) and focus group discussion (FGD) guides. The guides were developed by the research team following a thorough review of related literature. The data collection guides revolved around the participants' perceptions and experiences of PPE use in the workplace and their perceived impediments to PPE use. In addition, the guide had sections related to the workers' daily life in the laundry and the workers' vertical and horizontal relationships. The discussion guides used with both groups of the study participants were made similar to ensure the comparability of the responses obtained from them.

Inclusion in the FGDs was determined based on the study participants' homogeneity, convenience, and willingness. Key informant interviews were conducted in both hospitals alongside the focus group discussions. Personnel from Infection Prevention and Patient Safety Offices were the interview participants. Both the focus group discussions and the interview processes were held until the data reached saturation—a point at which recurrent patterns became evident in the participants' narratives. Apart from the study participants, the interview and FGD sessions consisted of a nonparticipant moderator/interviewer, a note-taker, and an observer. All the FGDs and the interviews were audio-recorded.

Open code software 4.03 was used for data analysis, which followed a thematic framework. First, the information gathered in the local language (i.e., Amharic) was transcribed verbatim. Later, the transcribed data was translated into English. The translation was made by an independent English language instructor. The data was analyzed after comparing the original transcript (i.e., the Amharic version) with its translated version (i.e., the English language version). This ensured the absence of discrepancies in words, meanings, and contents of the translated items.

Following this, the authors independently reviewed the transcripts before the process of sorting, coding, and theme identification. Next, data were validated and themes were developed based on an inductive and deductive process of issues that emerged from the discussions and the interviews. First, the authors developed themes independently. Later, the themes that could address the research questions evolved from the researchers' in-depth study of the individually developed themes. In presenting the data, relevant verbatim quotes are reported to aid the interpretation of the data.

Ethical clearance was obtained from the Institutional Review Board (IRB) of Hawassa University College of Medicine and Health Sciences. Before data collection, a permission letter was obtained from Hawassa City Health Bureau. Moreover, the participation of respondents was based on their full acceptance and volunteerism.

## 3. Result

Workers in the laundry departments were generally grouped into smaller units specializing in different tasks. Different activities are designated to the units in the department. For example, collecting dirty linens, washing dirty linens, drying, folding, storing, and distributing clean linens are among the tasks in the laundries of both hospitals. Altogether, 61 hospital workers participated in this study. Of these, 55 were laundry workers and six were personnel from Infection Prevention and Patient Safety Offices in the two hospitals. Participants in the FGDs were laundry workers while personnel from Infection Prevention and Patient Safety Offices were participants in the key informant interviews. Thirty-five (i.e., 53.0%) of the study participants were female. The mean age of the participants was 42.2 with SD ± 8.8 and range between18 and 56.

### 3.1. Barriers to PPE Use

#### 3.1.1. Themes and Subthemes (Categories) Identified

Two main themes emerged from the narrations of the participants regarding the barriers to PPE use: Organizational-level barriers and individual-level barriers. Within the theme of organizational-level barriers, two subthemes were identified: PPE unavailability and a disharmonious work environment. Within the theme of individual-level barriers, two subthemes were identified: low perception of susceptibility and belief about PPE interference with work performance (Table 1).

### 3.2. Organizational-Level Barriers

#### 3.2.1. PPE Unavailability

Implementing a PPE program is one of the most important strategies in hazard and risk management programs of hospitals [10]. However, during the focus group discussions, almost all of the participants described a general lack of PPE as a major barrier to their continued use of the full complement of PPE required for protecting them. The concern about decision-makers' lack of proper awareness about the hazards involved in the different tasks carried out within the laundries repeatedly raised reasons in several groups. Furthermore, evidence of hospital managers' failure to recognize and accept the role that timely, correctly, and sufficiently provided PPE can play in the control of the hazards was available in the FGD data. One respondent, for example, expressed her discontent with the scarcity in the supply of essential PPE items in the hospital as quoted below.

… Also, we do not have safety boots in this department. It is only given out to the maintenance department. We are not allowed to wear it because they tell us it is not given to the laundry staff. We have asked more than three times. (Hawassa FGD, Female 19 years)

The same respondent also described further the different tasks they often performed in the absence of PPE, exposing themselves and their families (at home) to health hazards.

We collect it [hospital linens], soak it with detergents, we wash it with a brush, place it in the dryer, and then fold it up. We do all this with no safety shoes on risking occupational accidents with puncture or laceration objects, especially needles, involving potentially contaminated biological materials. (Hawassa FGD, Female 19 years old)

In cases where PPE was available, not having them replaced regularly was also identified as a constraint to working with the full complement of PPE they needed.

I do not wear gloves because I do not have them and the reason I do not have them is that I cannot afford to risk my job by going to management to ask them to replace my torn gloves every other week. (Adare FGD, Female 38 years old)

Key informants considered in this study also had their share of discontent about the gaps in the continuous supply of PPE in the laundry departments.

…*We [hospitals] survive with our existing old gear because when we say the government provides equipment it does not mean it comes from a secret place filled with money. The government provides what it has and what it can and we the employees; we take what we can get and use it to the best of our advantage. (Adare hospital key informant)*

Many participants stated that besides insufficiency in the supply of items of PPE, the pieces of equipment that are currently in use are defective and poorly designed. The researchers endeavored much to understand the *defect* and the poor design of the PPE. Later, it came to be clear that the *defect* and the *poor design* referred to in the workers' dissatisfaction were the lack of durability of the pieces of PPE supplied to the workers.

…They give us boots, but it gets torn on the second day. So we walk on blood and other things that are washed out from the linen. … The gloves they give us get torn as soon as we hold washing brushes in our hands or carry a water container …. (Hawassa FGD, Male 30 years old)

Another key mention made was the issue of comfort or lack thereof felt by laundry workers when using PPE. Comfort is a nonstarter in the implementation of an effective PPE program [1]. However, the workers' testimonials show that discomfort experienced while using PPE was one of the reasons for failure to use PPE.

The boots that they give us burn us; they are also very heavy. The gown is not much different it does not fit well and is not comfortable when we move around and etc…. (Adare FGD, Male 22 years old)

A similar dissatisfaction, this time with respirators, was raised by another participant.

Although I know that we are supposed to use respirators while collecting dirty linens, I instead opt to cover my face with a scarf because the continued use of face masks has caused me skin damage (Hawassa FGD, Female 26)

Other than the problems with comfort and durability for some, these problems were compounded by working with ill-fitting equipment. Participants at various times reported that personal protective equipment such as overalls, boots, masks, gloves, and goggles was either too big or too small for them to work comfortably. Thus, it appears that the laundry workers had nothing much to use. Many largely appear to do their jobs with no protective equipment in the face of high-risk situations. One participant had the following to report in connection with the ugly choice she had due to ill-fitting gloves.

…We use heavy utility gloves for our work but I do not use them because it is hard for me to squeeze or fold linens with gloves that do not fit. The gloves that are procured by the hospitals are a one size fits all kind when they should be available in a variety of sizes. (Hawassa FGD, Female 33)

Data from key informants tends to agree with FGD participants' data.

One thing you have to know is we fill in the purchase orders for the best possible equipment for our workers. However, a lot of times we do not get what we order. Soon, we plan to get the funding to regulate the purchase and procurement of materials on site. (Adare hospital key informant)

### 3.3. A Disharmonious Work Environment

The data obtained in this study reveals the prevalence of unfriendly working situations in hospitals. For example, many participants in the study characterized the workload in the department as exhausting due to productivity demands. Others had the impression that they were working under constant pressure from managers. A significant number also expressed their dissatisfaction with what they called a “rigor control” from their immediate managers. In some cases, middle management representatives were also reported to have an unfriendly and authoritarian attitude toward workers. They said some personnel from the middle management appear to believe, perhaps wrongly, that the route to increasing and improving productivity and achieving goals in the workplace is through exhibiting an authoritarian attitude toward workers. This feeling of the reported “rigorous control” over workers' work pace and work break may have repercussions on the workers' health. In this regard, for example, many workers reported compromising between work pace and work safety.

… Workers are told not to change out of their protective clothing before going to the cafeteria because that would exceed the 20-minute tea break they have. So a lot of times we put on what we think are the most essential PPE like gloves only. Of course, we know that they [gloves] are also very easy to remove or put on in case we need to do it fast. (Hawassa FGD, Male 45 years old)

This frustration was shared by another worker who stated the following.

…We feel the pressure, we are responsible for everything and everything is our fault. We feel like any delay in our services may lead to serious problems for the patients. For instance, I usually work with no aprons or boots on because I feel that having them slows my movements affecting my output and getting me in trouble. (Hawassa FGD, Female 33 years old)

The absence of best practices in policy management was also discernable from the participants' testimonies. This had an impact on PPE use. For example, the condition of employment of a worker, i.e., whether one is employed on a permanent or a temporary basis, matters in terms of their getting PPE although both types of employees may have a similar exposure opportunity.

As you know, one of the devices that protect us against contamination is goggles, but getting goggles is difficult for workers employed under contractual terms. The supervisors often say goggles are for permanent employees. (Hawassa FGD, Female 22 years old)

The apparent mismatch between the participants' expectation to benefit from incentive programs and the reluctance of hospital administrators to set up relevant programs seems to affect the workers' enthusiasm for PPE use.

People who work hard are not appreciated here. I have many friends who work at Hawassa Pharmaceutical Industrial Park. They get bonuses and rewards like cell-phones in recognition of their best endeavors at the workplace. This should be implemented here to encourage safety and PPE use; noncompliance with PPE use should be punished and consistent use should be rewarded. (Hawassa FGD, Female 45 years old)

Alongside workplace policies, the unavailability of inventory tracking systems within the hospitals was identified as a factor that markedly impedes the workers' pursuit of PPE use.

… Nothing is certain in life but one thing that you can be sure of in this hospital is the constant PPE stock-outs. I do not think anyone monitors their usage or tracks their levels. (Adare, FGD, Male 33 years old)

The issue of training was also raised by the respondents as another important factor for the workers' failure to use any or some of the required protective pieces of equipment. The participants discussed the training they were given and expressed their dissatisfaction with the frequency (and the adequacy by implication) of the training. One participant, a linen collector was quoted stating the following:

They do give us training but in my opinion, it's not adequate. It is only given to us once a year, we got one training last year and the same thing happened this year,… I'd prefer it if it were at least once every 6 months. (Adare FGD, Male 30 years old)

The same participant explained why a training given only once in a year was not adequate.

In between the two pieces of training held one year apart many new hires come and go. So newcomers are usually left to their own devices to figure out why the equipment is needed and how and when they are used. (Adare FGD, Male 30 years old)

Key informants had their administrative views of the hospitals' training systems. They explained that running training frequently is expensive, given the expenses that go into organizing them more than once or twice in a year. The next excerpt illustrates this. The excerpt also reveals the informants' observation of incorrect worker behavior.

We are granted training budgets for only three days a year. We come prepared with slides and pictures to teach laundry workers how to wear gloves, masks, etc. Admittedly, we come across workers who find it difficult to remember lessons from the training and who do not respect hospital protocols. (Adare hospital key informant)

### 3.4. Individual-Level Barriers

#### 3.4.1. Low Perception of Susceptibility

In the group discussions, low susceptibility was mentioned as a reason for not using some or all of the recommended PPE during work. One reason in this regard was the service year in the workplace. Several workers, for example, who had long years of service in the department reported not using any PPE. They said they had enough experience performing tasks without getting sick or injured. For some, relying on experience and self-confidence when performing the work was most important in preventing infections; this is reflected in their positive perceptions of themselves and their conceptualizations of beneficial age-related changes such as the ability to carry out tasks with minimal risk to one's self. We found that overall older workers were more likely to view their late career more in terms of development than decline.

Experienced workers have mastered the tactics of working safely and avoiding any kind of danger, so I feel like I can function perfectly fine without it too. (Adare FGD, male 56 years old)

I have been involved in washing hospital linens for more than 15 years and I have never been injured, I know how to do the work safely regardless of PPE. (Hawassa FGD, Female 40 years old)

Likewise, other young participants generally agreed that more experienced workers are less likely to use PPE than their inexperienced counterparts. One participant linked this to the levels of a job promotion that come with experience as illustrated in the excerpt below.

Older and more experienced workers are more likely to be in positions of team leader or task supervisors and hence are usually involved in tasks that do not require them to wear PPE. (Hawassa FGD, Male 25 years old)

### 3.5. The Belief of PPE Interference with Work Performance

In the focus group discussions, the workers expressed concerns about the negative impact that using items of PPE might have on work performance. They were convinced that safety measures were a burden and an impediment to their ability to achieve productivity goals. Most understood the risks but assumed that they are capable of dealing with it to get the job done.

You feel like you're always adjusting it, and so it's hard to pay attention to what you're doing, when you're feeling that, that constant urge to fix it. (Adare FGD, Female, 27 years old)

Your speech gets muffled… so I have to repeat things to my colleagues. (Hawassa FGD, Female 29 years old)

## 4. Discussion

The level of personal protective equipment use among workers in two government laundry hospitals in Hawassa City, Ethiopia, was observed to be noticeably low. The impetus for this study arose from the recognition of the health risks associated with such a low level of PPE use in a hazardous work environment. This study was, therefore, designed to explore barriers to personal protective equipment use among laundry workers in the hospitals in Hawassa City. The data gathered and analyzed in response to the research concern shed light on some organizational- and individual-level barriers to laundry workers' use of PPE.

The scarcity of personal protective equipment in the hospitals was noted as a significant organizational-level barrier to the laundry workers' use of PPE at their workplace. This finding is similar to findings of other earlier studies conducted in India and the USA [5, 11]. In both studies referred to above, the absence of personal protective equipment was found to not only affect the adequacy and the quality of work but also endanger the lives and livelihoods of the laundry workers. In any health care setting, performing tasks effectively requires the provision of appropriate infrastructure and proper equipment and supplies [12]. The responsibility to ensure an adequate supply of PPE at the workplace lies with the employer [13].

Likewise, for effective PPE use, employees should be provided with equipment of an acceptable level of quality. This refers to equipment that can reduce physiologic burdens, improve communication, and be more comfortable and less of an encumbrance to wear. Otherwise, workers' commitment to PPE will be challenged [14]. In this study, lack of comfort, lack of fit, and lack of durability were found to put off workers from donning PPE. This finding confirms findings reported in studies conducted in China and Austria. In these studies, poor quality of PPE was found to affect the use of safety measures [15, 16].

Hospital policy may affect the use of and adherence to PPE [13, 17]. This was also found to be the case in this study. Workers' anger, depression, and hostility that emanated from their feelings about the administrative models in the hospitals acted as impediments to their PPE use in the workplace. The workers had the feeling that the administrative models in the hospitals were designed to ensure a technical focus on results rather than on people and their environment. This is indicative of the influence of the social environment on PPE use behaviors. It is known that a supportive safety climate in organizations positively affects the safety behavior of workers and should be part of the interventions necessary to improve PPE use [14].

The paramount need for a safe climate to prevail at the workplace was an issue of an in-depth discussion during the FGDs. For example, providing incentives for appropriate PPE use emerged during the FGDs as an example of the desirable safety climate of the workplace. However, incentive programs focusing on providing awards to employees solely for PPE use may only improve the employees' safety performance in the short term as they are unsustainable. On the other hand, incentive programs that promote safety awareness and employee participation in safety-related activities have been proven to be most effective [18]. Punishing noncompliance with PPE use protocol was another example of the desired safety climate of the workplace mentioned during FGDs. However, the motivational value of naming staff champions as role models has been proven to have the double benefit of increasing PPE use and workplace harmony [1].

In any health-related workplace, safety practices need to be sustained by a good level of knowledge and scientific evidence. The absence of this may lead to the spread of infections in the health care setting [19]. In this study, there was a noticeable difference between being aware of hazards, PPE, and PPE use. Several participants were overly confident and reported having a lower level of risk of acquiring infections compared to those who were younger and who had less experience. This is evidence of the existence of a clear gap in knowledge and attitude. In a study conducted in the United States, the phenomenon of long service years with no reports of workplace accidents was found to have led to a false sense of invulnerability, resultant noncompliance, and increased risk-taking [20].

Similarly, some participants, in the present study, exhibited misconceptions about PPE interference with performance. It is known that these types of beliefs result in increased risk-taking and ill-preparedness for the next unknown [20]. These beliefs, attitudes, and knowledge gaps can be changed, however, through scientific problem-based training programs. The determination of training needs and the establishment of separate training dimensions for knowledge about PPE, skills to use PPE properly, and attitude toward wearing PPE will be an important factor in the success of the training given to this working group over time. After all, workers in the laundry departments tend not to have adequate formal education in health care.

This study has some limitations. This is a two-site study, and the findings are not likely to be representative of other hospitals in their totality. Other hospitals will inevitably have their characteristics that mediate barriers to optimal PPE use, though it is probable that those identified in this study may have resonance there. Moreover, participant responses may be biased as a result of social desirability to provide sociably preferred answers. This means that opportunities for reluctance to reflect their real experiences among some participants cannot be ruled out. The outcomes reported in the present study are almost totally based on the perception of the study partakers, rather than hard evidence, e.g., tests of effectiveness, durability, and fit of protective clothing. The reported results have not been verified independently. Though perceptions are valuable, they can sometimes be colored by the enthusiasm and vested interests and, thus, may fail to accurately mirror actual circumstances as they exist. Also, the use of observations to monitor social dynamics and body language of participants during discussions could have been better approached through techniques that reduce observer biases such as video-observation. Aside from these limitations, the study possesses important strengths. The study provides a full and meaningful assessment of barriers to PPE use by including perspectives of laundry workers and infection prevention officers. The results add to a sparse body of the literature on barriers to PPE use among hospital laundry staff and can help future studies and interventions.

## 5. Future Research

Circumstances surrounding the ongoing Coronavirus (COVID-19) pandemic, together with very challenging working conditions, scarce resources, and stretched health care systems, make it difficult to collect data regarding the number of laundry workers dropping out because of infectious diseases possibly resulting from PPE nonuse. However, efforts to collect this data should be undertaken wherever possible. Similarly, an assessment of costs associated with PPE nonuse related injuries and worker and process downtime as a result of the injuries should be undertaken to better inform managers and policymakers. Future research would also benefit from a more structured sampling plan enabling the synthesis of larger sample sizes. In the future, studies related to PPE use among hospital laundry workers should incorporate designs that provide for carefully controlled intrahospital and interhospital comparisons over longer periods. The current study demonstrated that training is an important determinant of PPE use; nevertheless, it was examined in a general manner. Future studies may need to disaggregate it further and study the type and frequency of training, the content delivered, and the effect of the workers' prior knowledge on this training. Finally, one fairly narrow but an essential issue that struck the researchers' mind after their completion of the collection of the data used in the present study was related to the ethical implications of employing less educated and “underprivileged” members of a community in high-risk and infection-prone settings, particularly, settings like hospital laundries.

## 6. Conclusion

PPE nonuse was related to organizational- and individual-level factors such as PPE unavailability, workplace disharmony, low perception of susceptibility, and belief of PPE interference with work performance. Improving institutional supplies in quantity and quality may, therefore, have a positive implication for the improvement of infection prevention practices. Also, designing sustainable strategies and raising laundry workers' awareness of a safe work environment may lead to the improvement of PPE use. Workers, on their part, are expected to demonstrate personal responsibility for observing PPE protocol as needed at the workplace.

## Figures and Tables

**Table 1:** Barriers to PPE use among laundry staff of Government Hospitals in Hawassa, Ethiopia, April 2019.

Main themes	Subthemes (category)	Subcategories
Organizational-level barriers	PPE unavailability	Lack of ready access to full complement PPE in the workplace
		Poor quality of PPE:
		Lack of comfort
		Lack of durability
		Lack of fit
	A disharmonious work environment	Insufficient training
		Poor communication between workers and managers
		Productivity demands
		Lax control and monitor for PPE compliance
		Workplace policies
Individual-level barriers	The belief of PPE interference with work	Concerns of PPE interfering with work
	Low perception of susceptibility	Perceptions of beneficial age-related changes such as safe work practices
		Long service years without any recorded workplace injury

## Data Availability

The data sets used to support the findings of this study are available from the corresponding author upon request.

## Abbreviations

PPE: Personal protective equipment
HAIs: Health care acquired infections
FGDs: Focus group discussions
KII: Key informant interview.
